# Regulation of Cardiovascular Metabolism by Hormones and Growth Factors

**DOI:** 10.1155/2015/842351

**Published:** 2015-06-02

**Authors:** Cristian Ibarra, Sergio Lavandero, Manuel Estrada

**Affiliations:** ^1^Heart Failure Bioscience Department, Cardiovascular and Metabolic Diseases (CVMD), Innovative Medicines & Early Development (IMED) Biotech Unit, AstraZeneca R&D, 43150 Mölndal, Sweden; ^2^Advanced Center for Chronic Diseases (ACCDiS), Faculty of Chemical and Pharmaceutical Sciences, Faculty of Medicine, University of Chile, 8380492 Santiago, Chile; ^3^Department of Internal Medicine, Cardiology Division, University of Texas Southwestern Medical Center, Dallas, TX 75390-8573, USA; ^4^Programa de Fisiología y Biofísica, Facultad de Medicina, Universidad de Chile, 8389100 Santiago, Chile

Currently, metabolic disorders are a cardinal focus of research given their impact in public health problems worldwide. The main risk factors for death in people suffering metabolic disorders are cardiovascular diseases. Metabolic changes are important settings for cardiac cells under these conditions because the cardiovascular system must maintain energy balance to preserve work output and efficiency of the heart. Cardiovascular system is regulated by hormones which control, adjust, and remodel heart metabolism.

The role of hormones and growth factors in modulating cardiovascular function impacts different areas of research, especially considering that atypical plasma levels together with variable effects at cellular level are hallmarks of several medical conditions such as metabolic syndrome, obesity, diabetes, ischemic heart disease, and heart failure. Hormones and growth factors produce integrative systemic effects that involve ligand/receptor interactions, generations of second messengers, activation of intracellular signaling cascades, posttranslational modification of proteins, and gene expression. The implications of hormone and growth factor effects encompass the diagnosis, clinical evaluation, and pharmacological intervention. Several studies on metabolic effects of hormones and growth factors in the cardiovascular system are being translated into therapeutic applications.

In this issue E. Ostu et al. show the heart as a neuroendocrine gland that interacts with both hormones and cytokines. The authors discuss the current evidence about the dynamic communication between the heart and other organs involved in cardiovascular homeostasis. The exploration of microvascular function is important as predictive tool and as prognosis of cardiovascular risk and progression of heart failure. This paper describes the present knowledge on whether hormones and cytokines influence microvascular function and coronary flow reserve. The work is an interesting contribution to understand the pathological mechanisms of microvascular dysfunction which could further benefit patients suffering metabolic and cardiovascular diseases.

The incidence of cardiovascular mortality, including sudden death, is higher in men than in women. Obesity, metabolic syndrome, and type 2 diabetes mellitus are major risk factors for cardiovascular disease. Here, L. Skrgatic et al. review publications from the late 1980s to the present to infer whether polycystic ovary syndrome is a cardiometabolic risk factor according to its relationship to several intrinsic factors that are known to produce these metabolic disturbances. Meta-analysis and clinical evidence correlate low testosterone plasma concentrations with metabolic disorders and cardiovascular damage, but safety concerns have been raised over testosterone replacement therapy as an association between myocardial infarction and testosterone replacement therapy in men has been suggested. W. Reilly et al. carried out a large, retrospective study in a multicenter practice center for testosterone treatment. This analysis in patients treated with testosterone did not correlate with high incidence of myocardial infarction. Further studies should be conducted in this field in order to elaborate safe therapeutic replacement protocols.

Adipose tissue is an endocrine gland and key controller of body metabolism that interlinks with cardiovascular system. Early, simple, and fast biomarkers determining the association between altered focal point of adipose tissue and cardiovascular risk factors are useful for handling large scale and nutritional interventions and early therapeutic. The work of G. Curic et al. shows the relationship between subcutaneous and visceral adipose tissues with incidence of coronary artery disease in cohort of patients assessed at the cardiology unit. Using low cost anthropometric measures obtained from coronary artery disease and noncoronary artery disease patients, the authors found that anthropometric measures and subcutaneous adipose tissue have nonlinear relationship with the expansion of epicardial adipose tissue which is linked to coronary artery disease. Thus epicardial adipose tissue thickness and anthropometric measures have similar coronary artery disease predictive value.

Several studies have shown that consumption of energy drinks has short term impact on cardiac contractility. The cardiovascular and metabolic effects of these beverages have been associated with a high intake of caffeine, including high heart rate, palpitations, and increases in blood pressure, whereas hyperglycemic effects are associated with high sugar content. However, high concentrations of caffeine do not fully explain these effects; in this sense the paper of A. Panduric et al. explores the activation of the adrenergic system as a potential mechanism for cardiovascular and hyperglycemic effects produced by elevated energy drink intake. Through determination of heart rate, arterial blood pressure, blood glucose, adrenaline, and noradrenalin plasma levels before and after energy drink intake, the authors argue about positive cognitive functions and effect on cardiovascular and respiratory system at rest and during exercise by increasing activity of the sympathetic nervous system being due, in part, by adrenergic activation.

A large number of studies have shown that altered metabolic action of hormones and growth factors correlates significantly with the incidence of cardiovascular disease. These studies suggest the molecular basis for an integrated model of energy unbalance produced in cardiovascular system and hormone action and function. Furthermore, hormone interventional studies have shown an improvement in these cardiovascular risk factors.

*Cristian Ibarra* *Cristian Ibarra* *Sergio Lavandero* *Sergio Lavandero* *Manuel Estrada* *Manuel Estrada*

